# Public Perceptions and Information Needs of VCA Transplantation and Donation: A Mixed Methods Study

**DOI:** 10.3389/ti.2022.10752

**Published:** 2022-11-14

**Authors:** Alexander Ferzola, Jefferson Uriarte, Hannah C. Sung, Naomi Anderson, Carolyn Sidoti, Sarah E. Van Pilsum Rasmussen, Max Downey, Karen B. Vanterpool, Whitney Langlee, Samantha Klitenic, Lisa Young, Carisa M. Cooney, Ieesha Johnson, Allison Coleman, Jaimie T. Shores, Dorry L. Segev, Gerald Brandacher, Elisa J. Gordon, Macey L. Levan

**Affiliations:** ^1^ Department of Surgery, School of Medicine, Johns Hopkins Medicine, Baltimore, MD, United States; ^2^ Center for Health Services and Outcomes Research, Feinberg School of Medicine, Northwestern University, Chicago, IL, United States; ^3^ Department of Surgery, Grossman School of Medicine, New York University, New York, NY, United States; ^4^ Department of Plastic and Reconstructive Surgery, School of Medicine, Johns Hopkins Medicine, Baltimore, MD, United States; ^5^ The Living Legacy Foundation, Organ Procurement Organization, Halethorpe, MD, United States; ^6^ Department of Surgery, Feinberg School of Medicine, Northwestern University, Chicago, IL, United States

**Keywords:** vascularized composite allotransplantation, donation, perceptions, public education, focus group, qualitative, information needs

## Abstract

Vascularized Composite Allotransplantation (VCA) involves transplantation of multiple tissues from a donor to a recipient (e.g., skin, muscle, bone). Little is known about the US public’s perceptions of and attitudes toward VCA organ donation. This multi-site, cross-sectional, mixed methods study involved focus groups and surveys to assess members of the general public’s attitudes about VCA, and willingness and barriers to donate VCA organs. Qualitative data were analyzed by thematic analysis; quantitative data were analyzed by descriptive statistics. In focus groups (*n* = 6, 42 participants), most participants were female (57%) and Black (62%) with mean age of 42.6 years. Three main themes emerged: 1) awareness and perceptions of VCA, 2) purpose of VCA donation, 3) and barriers to VCA donation. Participants had heard little about VCA and sought information about VCA donation. Participants perceived VCA as challenging their concepts of “normality” and voiced concerns that VCA would create “Frankenstein[s].” Barriers to VCA donation included disruptions to end-of-life arrangements and information gaps regarding the donation process. Participants reported moderate to high willingness to donate their hands (69%) and face (50%) Public education efforts should address the specific needs and concerns of the public to facilitate VCA donation and family authorization.

## Graphical Abstract

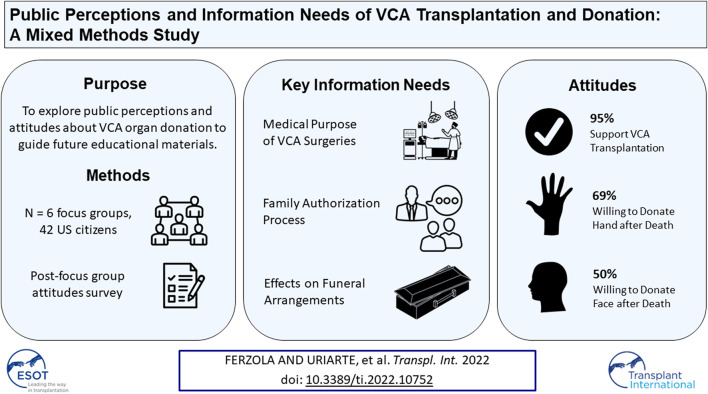

## Introduction

Vascularized Composite Allotransplantation (VCA) involves the transplantation of intact vascularized body parts, such as the hand, face, abdominal wall, and uterus, from a donor to a recipient (1, 2). VCA can potentially improve the quality of life for individuals who have suffered catastrophic traumatic injury, infection, and/or congenital anomalies (3). VCAs include the hand, upper extremity, face, uterus, penis, abdominal wall, and larynx. In 2014, the United States (US) Organ Procurement and Transplantation Network (OPTN) defined VCAs as organs, thereby applying the same regulatory status for policy development and allocation as solid organs within the country. There have been more than 100 VCAs performed in the United States since 1998, and over 165 VCAs have been performed worldwide (4, 5). Despite advances in the field, VCA authorization and subsequent donation rates in the US remain low, and information needs of the public regarding VCA transplantation and donation are little examined, which may help explain low prevalence of VCA (6).

Prior research reports little public awareness of VCA in the US and in other countries, but suggests a promising willingness to donate VCA organs once the public is minimally informed about VCA (7–9). Although the US media has featured several VCA-related human-interest pieces, including news stories about face transplants, VCA information in the public sphere has been limited, and more comprehensive educational materials about VCA transplantation and donation are needed (10). Due to the lack of educational materials and the prominence of popular culture ideals surrounding the purpose of VCA, the public may misunderstand or hold misconceptions about VCA. For example, public opinion surveys about face transplantation in the US and worldwide have reported a common belief in VCA’s purpose being primarily for cosmesis and psychological wellbeing rather than for functional use and survival benefits (7, 8, 11). Survey studies have found that public attitudes towards VCA are generally favorable, but may differ depending on the organ type (e.g., 53.8% willingness to donate a hand vs 39.0% willingness to donate a face) (7, 8, 12, 13).

Specific reasons for and insights into public willingness to donate VCA organs and barriers to VCA donation have been little examined, apart from perceived psychosocial benefits and risks regarding face transplantation (8, 9, 11). In addition, prior research on public attitudes about VCA has been based largely on surveys, and no research has qualitatively assessed the public’s perceptions and attitudes to gain in-depth insights into potential facilitators and barriers to VCA donation. Qualitative research is well-suited for examining group perceptions and elaborating on reported attitudes as well as identifying knowledge gaps in not well known topics, such as VCA.

Understanding public perceptions of and attitudes towards VCA can help identify knowledge gaps and concerns to address in order to foster public understanding and trust with VCA authorization and donation (14). Identifying knowledge gaps in the public’s understanding of VCA can reveal specific topics on which to provide information, common misconceptions to dispel, and barriers for donation to address. This paper assessed the public’s information needs, perceptions, and concerns about VCA to inform the development of educational materials to increase awareness of VCA donation.

## Materials and Methods

### Study Design

We conducted a multi-site, cross-sectional, mixed-methods study involving focus groups and surveys to assess the general public’s knowledge, perceptions, and willingness to donate or authorize VCA organs (15). A qualitative approach is useful for obtaining new, first-hand knowledge and descriptions about a phenomenon (16). Qualitative methods and results are reported in accordance with the Consolidated Criteria for Reporting Qualitative studies (17). Mixed methods enabled the elaboration and clarification of findings and increased validity of results (18).

### Setting and Participant Selection

The study was conducted at Johns Hopkins University (JHU) and Northwestern University (NU). Individuals were eligible for inclusion if they were English-speaking adults (>18 years) and US residents. Participants were recruited outside of Departments of Motor Vehicles (DMVs) in Baltimore, MD (*n* = 1 location) and Chicago, IL (*n* = 5 locations) between June and August 2019. DMVs offer excellent access to the general population for broad representation of the public. Research staff recruited interested individuals in-person by handing out flyers outside of DMVs and obtained their contact information for follow-up calls to schedule focus groups. Data collection occurred from June 2019 to December 2019. The Institutional Review Boards JHU (IRB00179535) and NU (STU00207605) granted approval. Participants provided written informed consent.

### Data Collection

We conducted *n* = 6 in-person focus groups (*n* = 3 focus groups at JHU in Baltimore, *n* = 3 focus groups at NU in Chicago), based on *a priori* goals for reaching thematic saturation (19, 20). Focus groups and surveys were conducted to assess public attitudes about VCA and inform subsequent development of VCA educational materials. A team of qualitative researchers and VCA experts developed the focus group moderator’s guide based on a prior content analysis of available public educational resources about VCA (10). The moderator’s guide was not pilot tested, but was reviewed by social scientists and clinical VCA experts to enhance face and content validity. Focus group questions assessed public perceptions, knowledge, and willingness to donate VCA organs. Focus groups were conducted by expert or trained focus group moderators (EJG, HCS, AF). Moderators used standardized guides to ask open-ended questions and encourage group participation (Supplementary File S1). Research assistants took hand-written field notes about the discussion and participant interactions. Before each focus group, the research team presented minimal information about the definition of VCA, types of VCA organs, and the definition of deceased donor to facilitate discussion. The research team answered participants’ questions related to relevant VCA discussion topics. Focus groups lasted approximately 60–120 min and were audio-recorded. Immediately following the focus groups, participants completed the paper attitudes survey in-person. The attitude items were adapted from a survey investigating attitudes toward VCA in metropolitan populations (8). The survey included closed-ended questions assessing support, willingness, and distaste for VCA using a 5-point Likert scale, and demographics (e.g., gender, age, race, education, marital status, employment, household income, health insurance, and prior experience with organ transplantation; Supplementary File S2, survey questionnaire). Participants were compensated $35 and $50 at NU and JHU, respectively, for their time.

### Qualitative Analysis

Audio recordings of focus groups were de-identified and transcribed verbatim. We analyzed transcripts using thematic analysis with both deductive and inductive coding (21, 22). Deductive codes were developed based on the questions asked during the focus groups. Inductive codes emerged for new topics during the focus groups (23). Transcripts were coded by four researchers (AF, HCS, NA, JU) trained in qualitative research methods by EJG, who has qualitative research expertise. Two researchers coded each transcript. Multiple rounds of coding with different coder pairs were conducted to establish inter-rater reliability (kappa ≥0.80). Differences in coding were reconciled by group consensus (24). After coding, we developed themes through writing code summaries to analyze common and disparate thematic concepts within each code segment across all focus groups and then compared thematic concepts across all codes. We used NVivo (12. lnk, QSR International Inc., Burlington, MA) for qualitative analysis.

### Quantitative Analysis

Descriptive statistics were performed on the post-focus group survey items assessing participants’ attitudes toward VCA. We calculated frequencies, means, and standard deviations (SDs) and compared attitudes by study site using Chi-squared and t-tests (*p*-value ≥ 0.05 was considered significant). We used Stata 17.0/MP for Linux (College Station, Texas).

## Results

### Demographics

Forty-two individuals (JHU: *n* = 15, NU: *n* = 27) participated in the focus groups (participation rate: 17%). Focus groups included, on average, 7 participants (range: 3–11). Most participants were female (57%), African American (62%), and had no prior experience with organ transplantation (69%). Participant demographic characteristics are detailed in Table 1. Sites differed demographically in terms of race/ethnicity, education level, employment status, and primary health insurance.

**TABLE 1:** Participants’ sociodemographic characteristics.

Variable	Total (N = 42) N (%)	JHU (*n* = 15) n (%)	NU (*n* = 27) n (%)	*p*-value
Age, mean [SD] (range)^a^	42.6 [14.2] (20–72)	45.1 [13.3] (24–62)	41.3 [14.5] (20–72)	0.33
Gender				
Female	24 (57.1)	6 (40.0)	18 (66.7)	0.12
Male	18 (42.9)	9 (60.0)	9 (33.3)	
Race/Ethnicity				
African American/Black	26 (61.9)	13 (86.7)	13 (48.1)	0.02^b^
White	9 (21.4)	2 (13.3)	7 (25.9)	0.45
Hispanic	5 (11.9)	0 (0.0)	5 (18.5)	0.14
Asian	4 (9.5)	0 (0.0)	4 (14.8)	0.28
Other	1 (3.7)	0 (0.0)	1 (3.7)	0.36
Marital Status				
Never married/single	19 (45.2)	6 (40.0)	13 (48.1)	0.31
Married/Domestic partner/Civil union	14 (33.3)	7 (46.7)	7 (25.9)	
Separated or Divorced	5 (11.9)	0 (0.0)	5 (18.5)	
Widowed	4 (9.5)	2 (13.3)	2 (7.4)	
Education				
Less than high school graduate	2 (4.8)	1 (6.7)	1 (3.7)	0.010
High school graduate	13 (31.0)	8 (53.3)	5 (18.5)	
Some college	14 (33.3)	6 (40.0)	8 (29.6)	
College graduate	8 (19.0)	0 (0.0)	8 (29.6)	
Post graduate degree	5 (11.9)	0 (0.0)	5 (18.5)	
Health Literacy (Help Needed for Reading Health Materials)^c^				
Adequate	37 (88.1)	15 (100)	22 (81.5)	0.18
Inadequate	5 (11.9)	0 (0.0)	5 (18.5)	
Employment Status				
Employed full-time	17 (40.5)	3 (20.0)	14 (51.9)	0.012
Not employed	12 (28.6)	8 (53.3)	4 (14.8)	
Retired	5 (11.9)	2 (13.3)	3 (11.1)	
Employed part-time	4 (9.5)	0 (0.0)	4 (14.8)	
Disabled	2 (4.8)	2 (13.3)	0 (0.0)	
Homemaker	1 (2.4)	0 (0.0)	1 (3.7)	
Student	1 (2.4)	0 (0.0)	1 (3.7)	
Income^d^				
<$15,000	13 (31.7)	6 (40.0)	7 (26.9)	0.21
$15,000-$34,999	12 (29.3)	6 (40.0)	6 (23.1)	
$35,000-$54,999	11 (26.8)	2 (13.3)	9 (34.6)	
$55,000-$74,999	3 (7.3)	0 (0.0)	3 (11.5)	
$75,000-$94,999	1 (2.4)	1 (6.7)	0 (0.0)	
$95,000+	1 (2.4)	0 (0.0)	1 (3.8)	
Primary health insurance				
Private	19 (46.3)	2 (13.3)	17 (65.4)	<0.001
Medicaid/Medicare	16 (39.0)	12 (80.0)	4 (15.4)	
None	4 (9.8)	1 (6.7)	3 (11.5)	
Other	2 (4.9)	0 (0.0)	2 (7.7)	
Registered Donor^e^				
Yes	21 (52.5)	8 (53.3)	13 (52.0)	1.00
No	19 (47.5)	7 (46.6)	12 (48.0)	
Experience with organ transplant				
Neither me nor anyone in my family has received a transplant or been on a transplant list	29 (70.7)	9 (60.0)	20 (76.9)	0.19
Not sure	5 (12.2)	4 (26.7)	1 (3.8)	
Someone in my family has received a transplant or been on a transplant list	4 (9.8)	0 (0.0)	4 (15.4)	
I have received a transplant or been on a transplant list	3 (7.3)	2 (13.3)	1 (3.8)	
Hours on the internet in a week				
I did not use the computer	3 (7.1)	1 (6.7)	2 (7.4)	0.95
Less than 5 h	6 (14.3)	3 (20.0)	3 (11.1)	
5–10 h	8 (19.0)	3 (20.0)	5 (18.5)	
10–15 h	8 (19.0)	2 (13.3)	6 (22.2)	
15–20 h	1 (2.4)	0 (0.0)	1 (3.7)	
More than 20 h	16 (38.1)	6 (40.0)	10 (37.0)	

^a^ JHU *n* = 1 not reported.

^b^ *p-*values measured across each race relative to each other.

^c^ Participants with responses “never,” and “rarely” were considered to have adequate health literacy. Responses of “sometimes,” “often,” and “always,” were considered to have inadequate health literacy.

^d^ NU *n* = 1 not reported.

^e^ NU *n* = 2 not reported.

### Focus Group Themes

Three main themes, or unifying concepts about subjects or meanings within the data (24), emerged from the focus groups: 1) awareness and perceptions of VCA, 2) VCA donation, 3) and barriers to donate VCA organs. Each theme comprised 3 or 4 sub-themes. Themes and corresponding representative excerpts can be found in Table 2.

**TABLE 2:** Representative excerpts by theme.

**1: Awareness and perceptions of VCA**	
1.1: Initial Perceptions of VCA	“It’s like the way things look has so much more of an impact on people even though people do not always say it does it does. And so I think that’s why there’s like this weird … it’s just like a little awkward because I do not even know if I could -- it would be just as weird to imagine your hand on someone else’s or like your face on someone else’s.” [Site 1, FG 2, Woman C]
1.2: Perceptions of VCA in Relation to Other Solid Organ Transplantation	“What would pop in my head if somebody told me that they had a [VCA] transplant? I do not know, I guess I would just look at them and say, ‘It looks good.’ Or maybe, ‘They messed you up.’” [Site 2, FG 2, Man A]
	“Is [VCA] important? I mean, I can see an internal organ. I mean, you’ll die. But if you live with one hand, you will not potentially die, and that’s what we’re hoping for, that nobody dies.” [Site 1, FG 1, Man A]
	“You can live without a uterus and you can live without a penis. So what’s the medical reason for somebody to have to get somebody else’s uterus or penis other than them wanting it? Is that what you were saying? … What’s the purpose of giving it to somebody?” [Site 2, FG 3, Woman A]
1.3: Questions about VCA	A comprehensive list of participant questions can be found in Table 3
**2: VCA Donation**	
2.1: Reasons to Donate VCA Organs	“I personally do not have any negative emotions toward [VCA] at all … It is [a] positive thing because I think it’s cool after you die, where you have one last thing to help however many people … and you can help that many people regardless of whether its extending life or just improving quality of life. Like that would be important to me, and I think that would be important to my family too.” [Site 1, FG 1, Woman C]
	“I’d probably just do it for the name of science. Just for the future, not necessary to save people, but just in the name of science, so they can further study and perfect it on how to do this with people in the future.” [Site 1, FG 3, Man A]
2.2: Willingness to Donate Hands and Face	“If I was a donor, I would donate my hand no problem, but not my face. Because that would be weird for my children. You know, they’re going to want to have a little funeral for me and even though I will not be here any longer, I just think that’s weird. The thing [s] that you gotta think [about face donation], and I will not donate. I do not care if I were getting cremated, I will not do it.” [Site 1, FG 2, Woman A]
	“I think I would be more okay with an organ than with a hand. I do not know, it does not matter but it just feels weird … But now, when I think hand or face, I feel different than internal organ. And I think it’s emotional … [Site 2, FG 1, Woman D]
2.3: Willingness to Donate Penis and Uterus	“Like, if I died, I would not want nobody getting my uterus … If it was just that they just wanted to have some children I do not agree with that.” [Site 2, FG 3, Woman A]
	“What if somebody comes in and just be like, ‘I want a sex change?‘… Because I feel as though, like myself, if I’m donating my body to help somebody, I do not want it to go to somebody that just wants their chemicals changed.” [Site 2, FG 2, Man D]
2.4: VCA Authorization	“And that person’s family, it do not matter if it resonate. If that person says what they want to do, it should be done.” [Site 1, FG 1, Male B]
	“I think that’s [VCA authorization] pointless because, if you already signed up for it when you were alive, and then somebody got to reauthorize it for when you dead or you’re about to die, … then it would be an issue.” [Site 2, FG 3, Female A]
	“Female F: They want it right away. They kept calling about my mother when she passed, like they want it right then and then. Like its no, you cannot … they cannot grieve
	Female D: Grieve
	Female F: They cannot wait that long. It has to be right away. So you have to make your mind up immediately
	Female D: That’s why she was saying, they all have to do that before they pass. You know, then it’s their decisions, your loved ones.” [Site 1, FG 3, Females D and F]
	“I think I would have to tell them, ‘When I go, you might see somebody that might look like me, might get my face, might get my hand, they might touch you and feel my --… I think that would be right, something that you can discuss with your family and your loved ones. It’s still your hand, your face, that’s a part of you, so if I’ve been around you for 70 years then I’m going to know your hands, I’m going to know your face. If I had to give this to somebody else to live, I would want somebody to expect that it might come up they might visualize me when I’m gone and they … they may have a trauma.” [Site 2, FG 1, Male A]
**3: Barriers to Donate**	
3.1: Religious and Cultural Beliefs	“Yeah. I see some difficulties when it comes to religion. And donating and different things because families have difficulties even dealing with whether their loved one want to be cremated or not or go the traditional route. That’s based on some religious beliefs. And, yeah, if religion is going to play a big part in whether the family or if the donor has not specified what they want to do other than being an organ donor, that will play a big part in whether families are willing to do that.” [Site 2, FG 3, Male 1]
3.2: Fear of Death	“Ok, well I think there’s going to be people rational or irrational that are going to have fears about well what’s really going to happen to my body parts? there’s just a lot of fear out there that is maybe unfounded that still rattles around and keeps people from donating.” [Site 1, FG 1, Female A]
3.3: Need to Improve Public Awareness of VCA	“Information is key, you know, every community if they’re not properly informed, their mind’s going to run wild with the idea of what could happen, what could be, so that’s what I think it comes down to is properly educating people.” [Site 2, FG 1, Man B]
	“I think the biggest issue is a lack of education, and a lack of awareness. And that people do not know that much about it. If they just knew just as much about [VCA] as they did about a heart transplant.” [Site 1, FG 2, Woman C]
3.4: Suggestions to Increase VCA Awareness	“The important information -- my opinion is it should be about saving lives … It should be mainly about the quality of their life and how donating these different parts of the body would or could affect someone else’s quality of life. They could live a little better or a little longer. I think that that should be stated a lot that would help [Site 1, FG 3, Man A]
	“Write about real life experiences. People that have gone through the process, received a hand or hands and face and how their life was improved.” [Site 2, FG 1, Man A]

#### Awareness and Perceptions of VCA

Most participants reported being unfamiliar with VCA. Participants discussed their initial perceptions of VCA, compared VCA to solid organ donation, and asked questions about a variety of VCA topics.

##### Initial Perceptions of VCA

Most participants across all focus groups had never heard of VCA prior to study recruitment. While participants had not heard of the term “VCA”, some participants recalled hearing about face and hand transplants through major news outlets and newspaper articles. Participants associated VCA, particularly face transplants, with popular culture references including the television show “Game of Thrones” and the movie “Face Off.” Participants perceived VCA as a procedure from fantasy or science fiction and commented about the potential of VCA to create “cyborgs,” “clones,” or “Frankenstein[s].” Accordingly, they expressed concerns that as VCA evolved, it may push the boundaries of “normality.” Furthermore, participants perceived VCA as “weird” or strange to imagine “your face on someone else’s [face/body].”

##### Perceptions of VCA in Relation to Other Solid Organ Transplantation

As VCA was an unfamiliar topic, participants used their knowledge of the more familiar solid organ transplants (e.g., liver, kidney, and heart transplantation) to ask about or note similarities and differences compared to VCA. Participants described solid organs as “internal,” while they classified VCA organs as “external” because people can visualize it or “see how it looks.” When discussing “external” organs such as hands or faces, discussions focused on the appearance of the donated VCA organ on its recipient after surgery.

Participants viewed “internal” organ transplantation as vital or lifesaving, but questioned the medical “purpose” or necessity of VCA, specifically VCAs such as uterus and penis. They also questioned if the potential benefits of VCA would outweigh the risks to its recipients (e.g., side effects, medical complications, immunosuppression drugs).

##### Questions About VCA

Overall, participants asked 208 questions about VCA during focus group discussions, reflecting their information needs. Participants asked about numerous topics including the history of VCA, potential VCA recipients, outcomes of VCA recipients, and the processes for donating and for receiving VCAs. Regarding the relationship between VCA donors and recipients, participants desired clarification on how donors and recipients are matched for skin color and size, if recipients would appear exactly like their donor, and if the recipient’s new appearance would create legal identification issues (e.g., identification photos, fingerprints). A comprehensive list of participant questions can be found in Table 3.

**TABLE 3:** Representative participant questions.

**Success and outcomes of VCA (*n* = 6 focus groups)**
All of [VCA] surgeries been a success?
What’s the percentages of the [VCA] completely working? What’s the percentages of the failure?
Has there been a time you have attached the hand and had to remove it because it just did not work?
Will you function normally [after VCA]?
Suppose the hands or the upper limbs, they get done and everything but they’re not successful. Do they redo it and try to connect it and find the problem? Or that’s it for you?
What was the success rate?
How did the [recipients] accept it? You know, how’s their mental state?
Another concern like going back to psychological aspects, how—a person’s face is almost integral to who they are as a person. How much does the face transplant affect their appearance to the point where they become indistinguishable from who they used to be? Like how successful is that?
Are they still working the same for them? How my hands working when I had, how do they actually feel you know. What type of joy would it bring to them after they have received it?
**VCA Surgery Process (*n* = 6 focus groups)**
When you do the kidneys or when you do the blood transfusion, with the hand or the face, do you still have to have that same blood type?
Or is [VCA] like a graft again where they take a part and try to grow it or--?
With the face transplants is it the full face or do they just get parts of their face transplanted?
With these transplants right here, like the uterus and the penis, so when they transplant, do they transplant the full uterus, and the full penis? Or is it partial?
**History of VCA (*n* = 5 focus groups)**
Where was the first VCA performed?
Are they doing it in the States?
Now how long have they been doing this procedure, the VCAs?
Is this something being done now or are you talking futuristic?
**Timeline of VCA Process (*n* = 5 focus groups)**
How long does the process take for the surgeries and everything? You said you’ve got to match and do the blood and all that. Like, how long would we be waiting?
How long is the recovery?
How lengthy is the process, like donor as well as recipient, to have to fill out complete paperwork? Is it hard or is it easy?
Like the process of rehabilitation, do you have to go through the same process with that transplanted arm or limb, just like if you were to rehabilitate yourself? Would it be like the same process you have to work that hand out or limbs out the same way?
**Appearance of VCA Organs (*n* = 5 focus groups)**
When they say face transplant it’s like you completely change it?
How would the face and everything, how would they get you to look close to that skin or something like that?
Will [the donated face] be the exact same look as me?
Does there have to be some kind of compatibility? Like, small versus large, women versus men?
Now do they match color and color?
**Cost of VCA (*n* = 5 focus groups)**
Well who will pay for that, insurance would not pay for that, right?
Okay say if it was me and I needed one of these VCA transplants, I would not even be able to afford it because I looked it up and they cost four, five million dollars for some of these so how would that work out for me?
Would insurance cover it or you got to pay for it in cash?
**Potential VCA Recipients (*n* = 4 focus groups)**
So [VCA] would only be just for soldiers and veterans?
Was it just a regular person that got it done?
Would [VCA] just be for the other people that can afford it?
With this transplant, does age have anything to do with it? Do you have to be 18 and over or 21 and over to be qualified to do transplant? Or can it be a child?
So can it be used for people who have been severely burned, third degree burns?
**Becoming VCA Donors (*n* = 4 focus groups)**
So you can pick [which organs to donate], you can be like, “Okay, you can take their hand or their foot”?
VCA is going to be added to the Motor Vehicles if people want to donate this … ?
Will it become like a part of like a contract where you know when you go to the hospital and you sign the waiver about being treated and everything, will you start putting that into the form, too, like if you want if something happens, would you want your body donated? Would that start becoming inside of that contract?
The question on our driver’s license, it’s just are you a donor, yes or no … did it always imply every part of your body?
You know how you sign up to be an organ donor? You’ve got to actually go and sign up to be a VCA donor? Or is it all composited into one?
**Family Authorization (*n* = 3 focus groups)**
Say if somebody is an organ donor and they got one of those “do not resuscitate” orders do they still ask their family for their organs?
So even though you signed off to be an organ donor your family still got to agree with it at the end?
Even if you do not sign off on it, your family will still have to agree at the end anyway, right?
Would you ask [the family] right away when they die, or would you wait? You know, cause some people are grieving, and they get angry, and they’ll be like, “No!” Would you ask right away, or would you wait?
**Religious/Cultural Concerns (*n* = 3 focus groups)**
Have you surveyed any other religions, and know which ones would be the ones that say no?
Do they include religion to it as a factor when they go to pick [VCA organs]?
They just do not consider religion and lifestyle? If it’s the same blood type then you’re getting it. That’s how it goes pretty much?
**Funeral Concerns (*n* = 3 focus groups)**
When do they take off the face and the hand? After the funeral?
How soon after the person like dies would you take their hands and face?
Will you still be able to have a funeral?
**Association of VCA with other medically-related procedures (*n* = 2 focus groups)**
Is it like I can just call and say, “Hey, I want to do this” like plastic surgery?
What if somebody comes in and just be like, “I want a sex change?”
If this is at all possible, then we’re talking about possibly clones. Are we going that far?

#### VCA Donation

Across focus groups, participants discussed reasons that they were more or less willing to donate or authorize VCA organs. Some participants expressed that they were willing to donate all VCA organs, while others provided reasons for being unwilling to donate specific VCA organs. Participants sought clarification on the VCA donation and authorization processes.

In the post-focus group surveys, nearly all participants (95%) reported that they “strongly agreed” or “agreed” that they were in support of VCA transplantation (Table 4). Furthermore, only a few individuals (4%) reported that VCA transplantation was distasteful to them.

**TABLE 4:** Participant attitudes about VCA.

Factor		N^a^	Strongly disagree n (%)	Disagree n (%)	Neutral/Unsure n (%)	Agree n (%)	Strongly agree n (%)	*p-*value^b^
I support VCA transplantation	Total	42	0 (0)	0 (0)	2 (5)	16 (38)	24 (57)	0.10
	Northwestern	27	0 (0)	0 (0)	2 (7)	7 (26)	18 (67)	
	Johns Hopkins	15	0 (0)	0 (0)	0 (0)	9 (60)	6 (40)	
I would be willing to donate my hand upon death	Total	42	0 (0)	2 (5)	11 (26)	12 (29)	17 (40)	<0.001
	Northwestern	27	0 (0)	1 (4)	9 (33)	2 (7)	15 (56)	
	Johns Hopkins	15	0 (0)	1 (7)	2 (13)	10 (67)	2 (13)	
I would be willing to donate my face upon death	Total	40	2 (5)	5 (12)	13 (32)	6 (15)	14 (35)	0.09
	Northwestern	26	2 (8)	2 (8)	8 (31)	2 (8)	12 (46)	
	Johns Hopkins	14	0 (0)	3 (21)	5 (36)	4 (29)	2 (14)	
I would be willing to receive a hand transplant after a severely deforming accident	Total	42	0 (0)	0 (0)	10 (24)	21 (50)	11 (26)	0.70
	Northwestern	27	0 (0)	0 (0)	7 (26)	12 (44)	8 (30)	
	Johns Hopkins	15	0 (0)	0 (0)	3 (20)	9 (60)	3 (20)	
I would be willing to receive a face transplant after a severely deforming accident	Total	41	1 (2)	1 (2)	14 (34)	18 (44)	7 (17)	0.30
	Northwestern	26	1 (4)	0 (0)	7 (27)	12 (46)	6 (23)	
	Johns Hopkins	15	0 (0)	1 (7)	7 (47)	6 (40)	1 (7)	
VCA is distasteful to me	Total	42	17 (40)	16 (38)	7 (17)	1 (2)	1 (2)	0.20
	Northwestern	27	12 (44)	7 (26)	6 (22)	1 (4)	1 (4)	
	Johns Hopkins	15	5 (33)	9 (60)	1 (7)	0 (0)	0 (0)	

For understanding participant attitudes from the post-focus group survey, agree and strongly agree were combined for general favorability.

^a^ N refers to number of respondents to each question.

^b^ *p-*value measured for differences between research sites; missing data was treated as a separate variable.

##### Reasons to Donate VCA Organs

Participants willing to donate any VCA organ noted their perceived benefits of VCA were to “help a lot of people” and to improve the quality of life of its recipients. Participants proposed that burn victims, people from the military, and people with “defects” could potentially benefit from being recipients of VCA. Participants reported that they would feel comfortable donating VCA organs to a recipient who had undergone “something traumatic” and who would “use it wisely,” but they would not donate to someone who only wanted to pursue VCA for plastic surgery. Some participants reported being amenable to donating VCA organs because they perceived VCA donation to be a similar concept to solid organ donation. Other participants expressed that they would be willing to donate VCA organs “for the name of science,” or in order to advance the field.

##### Willingness to Donate Hands and Face

Participant’s comments suggested mixed opinions and hesitation or concern about donating hands and faces. Compared to donating solid organs, participants perceived hand or face to be “weird” and “emotional,” as the hands and face are more closely related to appearance and personal identity. Participants did not want to donate their own hands and/or face because they did not want family members to feel uncomfortable during funerals. Participants were also concerned that family members could experience emotional “trauma” from seeing their loved one’s organs on the recipient’s body.

Despite expressed concerns about hand and face donation, participants reported moderate to high willingness to donate their hands (69%) and face (50%). Participants were more willing to receive hands (76%) or a face (61%) than to donate these organs (Table 4).

##### Willingness to Donate Penis and Uterus

Participants expressed strong views about uterus and penis transplants. They reported not being willing to donate a uterus or penis if prospective recipients wanted to have “a sex change” because these motivations went against participants’ religious and personal beliefs. Comments about changing bodies and genders sparked heated discussion between participants as they disagreed whether there should be “stipulations” or non-medically related eligibility criteria to receive VCA.

##### VCA Authorization

Participants expressed confusion about the authorization process for VCA donation in the US because it requires a next-of-kin or family authorization after the registered donor’s death, which is not required for solid organ donation. Some participants who were registered donors believed that they had already authorized VCA donation, and thus were confused about family authorization. After clarifying the VCA authorization process, some participants stated that there was “no point in signing up” as a VCA organ donor because the family and/or next of kin “will still have to agree” to the donation decision. Participants discussed the importance of interested potential VCA donors to speak with their families about their desire to become VCA donors and agreed that the family or next of kin should “respect” and concede to the individual’s “wishes” to donate VCA organs.

Participants expressed several concerns for their families in making VCA authorization decisions. Participants discussed the burden placed on families who would have to make authorization decisions quickly to ensure VCA organs remain viable for transplantation. Participants feared that family members would not have ample time to “grieve” the death of their loved ones. Participants agreed that families should have discussions about VCA donation wishes to prepare for the burden of decision making and seeing their loved one’s VCA organs on another individual.

#### Barriers to Donate

Across focus groups, participants discussed potential barriers to VCA donation which included: religious and/or cultural beliefs, fear arising from thoughts about death, and lack of information and awareness of VCA donation. Further, participants made suggestions on how to increase public knowledge and awareness of VCA donation to address potential barriers.

##### Religious and Cultural Beliefs

Participants commented that VCA donation might violate religious and cultural beliefs and interfere with the donor’s plans “to have an open casket” funeral, especially after face donation. Participants recognized that individuals from various religious and cultural backgrounds may want to keep their bodies intact after death. Some participants commented that the organ procurement process might inhibit family member’s ability to “grieve” for their loved one before an organ procurement agent approaches them to make an authorization decision.

##### Fear of Death

Participants discussed the visceral or “irrational” fear that the public may experience when they first hear about VCA donation. Participants stated that fear could arise from associating VCA donation with death and imagining their body parts, including their limbs and faces, being removed. Furthermore, participants recognized that people may fear VCA because of its relative newness compared to solid organ transplantation and the lack of knowledge and awareness about VCA donation among the public.

##### Need to Improve Public Awareness of VCA

Focus group participants stated that the lack of information and awareness about VCA would prevent the public from donating VCA organs. Participants acknowledged that people may be misinformed and possess “incorrect ideas” about VCA donation and its purpose. Participants suggested that the lack of awareness surrounding VCA could be addressed through education.

##### Suggestions to Increase VCA Awareness

Participants recognized the importance of educating the public about VCA to increase awareness. Participants recommended including a description and purpose of VCA in educational materials, such as clarifying that VCA is for medical rather than cosmetic reasons to improve a person’s quality of life. Because many participants were learning about VCA for the first time, they suggested explaining the acronym “VCA” and making the term VCA understandable. Participants agreed that public educational materials should be comprehensive and describe risks, side effects, and outcomes. Participants explained that providing clear information about the pros and cons of VCA could help potential recipients and donors make informed decisions. Further, participants suggested including VCA success stories and recipient testimonials to make VCA more relatable or appealing to the general public.

Participants also recommended different types of informational modalities to educate the public. Participants suggested the use of social media and advised sharing educational materials in public locations that typically engage large numbers of people (e.g., train stations and bus stops). Participants mentioned targeting education campaigns to potential audiences who could benefit most from VCA educational materials, specifically students and healthcare workers. Overall, participants recommended making information accessible, comprehensive, and relatable to the public.

## Discussion

Our qualitative study of public attitudes about VCA in the US found that while participants were generally unaware of VCA, they may be willing to donate certain VCA organs after being informed about VCA and they may possess certain religious/cultural beliefs that prevent them from donating. Participants’ information needs about and barriers to VCA donation should be addressed through educational materials to help increase awareness and accurate knowledge of VCA, its purpose, and the authorization and donation process.

Participants’ impressions of VCA pertained to misrepresentations and/or misconceptions about VCA likely due to a lack of awareness about the procedure and the information presented in the public sphere through popular television shows and movies. Media and popular culture influences the daily lives of the public, which affects how and what people think about themselves and others, including personal and social issues (25). In addition, prior media coverage of VCA and organ transplantation in general has tended to promote stories that are “sensational” rather than strictly for educational purposes (10). Thus, raising awareness and properly educating the public about VCA may help to address misinformation spread through media and to foster understanding about the purpose of VCA transplantation and donation.

Participants, through focus groups and surveys, reported varying levels of comfort in supporting specific VCA organs. Our focus group and survey findings corroborate previous survey studies from around the world that reported less willingness to donate VCA organs than solid organs (kidney, liver, heart, lungs) and greater willingness to donate hands than the face, penis, or uterus (8, 12, 13). In addition, qualitative insights from focus groups corroborate reasons for lower willingness to donate the face, including not wanting to donate in order to retain one’s identity and bodily integrity after death and to allow for their family’s grieving (8, 11). Moreover, our focus group study found that participants in the two US metropolitan areas sampled from might be unwilling to donate a uterus or penis to a recipient who desired to alter their sex, which contrasts with a US survey study reporting 69.3% public willingness to donate a uterus or penis to an individual of a different sex (13). To our knowledge, no VCAs have been performed for the purpose of transgender sex changes to date.

Participants expressed confusion about the authorization process to become a VCA donor in the US and how this differs from solid organ donation. Participants viewed donation as complicated mostly because individuals were unaware of the proper procedure(s) of becoming a VCA donor. Many participants believed that once an individual becomes a registered donor through the Department of Motor Vehicles, they are authorizing VCA donation in addition to authorizing donation for other solid organs. Moreover, because VCA authorization occurs quickly after the death of the potential donor and is provided by the next of kin, VCA authorization may become a burden for family members dealing with grief and funeral planning. VCA educational materials should address confusion regarding VCA authorization by explaining the steps needed to become a VCA donor. Such information will educate and better enable individuals to engage in conversations with family members to express their desire to become a VCA donor and help family members prepare for next-of-kin authorization.

Participants recommended making information accessible, comprehensible, and relatable to increase public knowledge and awareness of VCA. Our prior content analysis of existing educational materials for VCA, including materials from OPOs, transplant centers, OPTN, and the Department of Defense, revealed that most materials referenced a specific story (75%), some materials described potential benefits (15%), and few mentioned the appearance of a transplanted VCA organ (1%) (10). While pre-existing materials were relatable by describing specific case studies of individuals who the public can see and feel empathy for, materials did not address topics such as the difference between VCA and other solid organs, VCA authorization and donation processes, and culturally specific burial customs which were topics of discussion in focus groups. By addressing these information gaps and concerns, educational materials may increase the public’s awareness and understanding of VCA and help ameliorate concerns about VCA donation.

Educational materials should address participants’ most prevalent information needs, such as describing VCA outcomes transparently, understanding the VCA evaluation and surgical process, information on the state of VCA, and dispelling misconceptions such as appearance modification after transplant. Through educational materials, we may also begin to address concerns that individuals hold about family donation and the cultural and/or religious barriers to donation.

Our study has several strengths. We conducted focus groups at multiple sites located in large, geographically distinct US cities, with participants representing diverse backgrounds, which increases the transferability and generalizability of our findings. A limitation of this study is that participant statements and attitudes towards VCA may not reflect actual behaviors. We recruited from urban and suburban DMVs, and thus findings may not be generalizable to rural populations (26). With the results of this study outlining the major barriers and concerns about VCA and VCA donation, future research should leverage study findings to inform the development of educational materials, then assess whether implementation of educational interventions with a culturally competent focus can contribute to an increase in positive public perceptions of VCA and VCA donation rates.

## Conclusion

Our study assessed the public’s knowledge, perceptions, and willingness to donate VCA organs to inform the development of educational materials to increase awareness of VCA donation. Study findings revealed that although the general public may have concerns and information needs about VCA donation, willingness to donate VCA organs is generally favorable. Public education should address the specific information needs and concerns outlined by members of the public in order to better prepare the public to become VCA donors and/or authorize VCA donation.

## Data Availability

The datasets presented in this article are not readily available because the raw data are proprietary and might be used to author future publications. Requests to access the datasets should be directed to the corresponding author.

## Abbreviations

DMV, department of motor vehicles; JHU, Johns Hopkins University; NU, Northwestern University; OPTN, organ procurement and transplantation network; US, United States; VCA, vascularized composite allotransplantation; VCAs, vascularized composite allografts.
