# Supplementary material for: Public Perceptions and Information Needs of VCA Transplantation and Donation: A Mixed Methods Study
Source: Transpl Int. 2022 Nov 14;35:10752. doi: 10.3389/ti.2022.10752 (PMC9701711; doi:10.3389/ti.2022.10752)
Supplement: Supplementary file 1 [file DataSheet2.pdf]

FG # ____ Date: __/__/____ ID # ____________

DOD VCA- Post Focus Group Survey

The purpose of this brief survey is to get your impression of the focus group overall and to ask some background questions. Your responses will be anonymous. Please respond to questions in as much detail as possible. (Use the reverse side for more space).

1. Were there some things that you had not been willing to say in the group discussion?

___ No

___ Yes → Please specify what you would have liked to say:

2. Were there any questions that should have been asked?

___ No

___ Yes → Please specify questions:

3. Please reflect on the discussion. Do you have any further suggestions about what information or topics that we should include in the educational materials about VCA transplantation? Please describe here:

Please specify how much you agree or disagree with the following statements:

**4.**	I support VCA transplantation.	Strongly Agree	Agree	Neutral/Unsure	Disagree	Strongly Disagree
**5.**	I would be willing to donate my hand upon death.*	Strongly Agree	Agree	Neutral/Unsure	Disagree	Strongly Disagree
**6.**	I would be willing to donate my face upon death.*	Strongly Agree	Agree	Neutral/Unsure	Disagree	Strongly Disagree
**7.**	I would be willing to receive a hand transplant after a severely deforming accident.*	Strongly Agree	Agree	Neutral/Unsure	Disagree	Strongly Disagree
**8.**	I would be willing to receive a face transplant after a severely deforming accident.*	Strongly Agree	Agree	Neutral/Unsure	Disagree	Strongly Disagree

*Source: Sarwer et al. Vascularized Composite Allotransplantation 2014;1(1-2):22-30.

9. Are you registered as an organ donor (e.g., on your driver's license or on a state or national registry)?

___ Yes

___ No

Please continue to the next page →

10. Last week how many hours did you spend using the Internet?

___ I didn't use the computer
___ Less than 5 hours
___ 5-10 hours
___ 10-15 hours
___ 15-20 hours
___ More than 20 hours

Demographics

Please help us learn a little bit more about you by responding to these last few questions. This information will be kept confidential.

1. Are you: ☐ Male ☐ Female ☐ Other

2. How old are you? ________ years

3. What is your race (check all that apply)?

☐ White
☐ Black or African American
☐ American Indian or Alaska Native
☐ Hispanic or Latino
☐ Native Hawaiian or Other Pacific Islander
☐ Asian
☐ Multi-racial
☐ Other (please specify): _________________________

4. What is the highest grade or level of formal education you have completed?

☐ Less than high school graduate
☐ High school graduate
☐ Some college
☐ College graduate
☐ Post graduate degree (MA, PhD, MD, DO, etc.)

5. What is your present marital status?

☐ Married/Domestic partner/Civil union
☐ Separated or divorced
☐ Widowed
☐ Living with partner
☐ Never married/single

6. What is your current employment status? *Please check one.*

☐ Employed full-time
☐ Employed part-time
☐ Not employed
☐ Homemaker
☐ Retired
☐ Disabled
☐ Student

Please continue to the next page →

7. What is your total household income for the past 12 months (you + spouse/partner)?

☐ Less than $15,000
☐ Between $15,000 and $34,999
☐ Between $35,000 and $54,999
☐ Between $55,000 and $74,999
☐ Between $75,000 and $94,999
☐ More than $95,000

8. What primary health insurance do you have?

☐ Private health insurance (through employer or self-pay)
☐ Medicaid/Medicare
☐ None
☐ Other: ____________________________

9. Have you or anyone in your family ever received an organ transplant, or been on a transplant list?* (*Source: Sarwer 2014)

☐ Yes, I have received a medical transplant or been on a transplant list
☐ Yes, someone in my family has received a medical transplant or been on a transplant list
☐ No, neither me nor anyone in my family has received a medical transplant or been on a transplant list
☐ Not sure

10. How often do you need to have someone help you when you read instructions, pamphlets, or other written material from your doctor or pharmacy?

___ Never
___ Rarely
___ Sometimes
___ Often
___ Always

- **Please hand your completed survey to Elisa Gordon.**
- **After we receive your survey, we will compensate your for your time.**
- **If you have any questions please contact [name] at 312-503-xxxx.**

Thank you very much for your time!
