# Supplementary material for: Public Perceptions and Information Needs of VCA Transplantation and Donation: A Mixed Methods Study
Source: Transpl Int. 2022 Nov 14;35:10752. doi: 10.3389/ti.2022.10752 (PMC9701711; doi:10.3389/ti.2022.10752)
Supplement: Supplementary file 2 [file DataSheet1.pdf]

Focus Group Moderator Guide

Welcome to this group discussion. My name is [insert name] and I am the moderator. My role is to help get a conversation going and to make sure we cover a number of important topics that we would like your input on.

<u>Purpose:</u>

First of all, I would like to thank you for taking the time out of your day to come here and discuss your ideas. The overall goal is to hear your thoughts on hand, arm, or face transplants, and your thoughts on educational materials that provide more information about these types of transplants.

I'd like to tell you about the research study we are going to discuss. [Now share the aims of the study and the purpose of the FG and what you need from participants.]

Before we begin, I want to make clear that:

- You are the experts and we are here to learn from you.
- Participating in this discussion is strictly voluntary.
- [Insert name] is here to assist me and will be taking some notes once our discussion gets started. We would also like to audio record what you say so that we don't miss anything important and so that we can go back and revisit the information if we need to.
- We expect our meeting to last up to 2 hours

As far as focus groups are concerned, there are a few "ground rules" we ask your help with.

- I might move you along in conversation. Since we have limited time, I'll ask that questions or comments off the topic be answered after the focus group session.
- I'd like to hear everyone speak so I might ask people who have not spoken to comment.
- Please respect each other's opinions. There's no right or wrong answer to the questions I will ask. We want to hear what each of you think and it's ok to have different opinions.
- We'd like to stress that we want to keep the sessions confidential so we ask that you not use names or anything directly identifying when you talk about your personal experiences. We also ask that you not discuss other participants' responses outside of the discussion. However, because this is in a group setting, the other individuals participating will know your responses to the questions and we cannot guarantee that they will not discuss your responses outside of the focus group.
- I will refer to whatever name you want me to call you. If you would like to use another name other than your own this is fine. Please write the name you want me to use on your name tag.

Do you have any questions so far?

Before we begin, we'll do a quick **icebreaker**.

> Tell us a little something about yourself. Examples: how long have you lived in the Baltimore area, what do you like to do, or do you have any celebrations you would like to share?

VCA Public Education Focus Group Questions

VCA stands for Vascular Composite Allotransplantation. Examples of VCA include hand, arm, or face transplants. [Have the full name of VCA and examples of VCA written on a poster or board.] In VCA, a recipient would receive a hand, arm or face transplant from a deceased donor, someone who has passed away.

1. Has anyone heard of VCA before?
    a. If so, where have you heard it?
2. Based on what you've heard before or what you've learned today, what do you think about VCA?
3. What words come to mind when someone says hand, arm, face… [*reference poster or board*] transplant?
4. Is anyone here a registered donor?
    a. For those who are not currently registered donors…
        i. How do you feel about organ donation?
        ii. Are there any reasons you decided not to become a donor?
            1. What are some of your hesitations or doubts about donation?
        iii. How do you feel about hand, arm, or face donation or transplantation?
    b. For those who are registered donors…
        i. What are some reasons you decided to become a donor?
            1. Do you have any hesitations or doubts about being a donor?
        ii. How do you feel about the idea of being a hand, arm, or face… [*reference poster or board*] donor?
            1. How is it the same or different from donating internal organs, such as kidney or liver?
            2. If you decide to be a VCA donor, what conversations do you imagine you will need to have with family, friends, or others?
            3. Would you give permission/authorization to your family to be a VCA donor?
5. What information do you think should be included in education materials about VCA?
6. What are some ways we can educate the public about VCA (types of education materials, where to post education materials, who receives education materials)?

7. What may be some personal, financial, cultural, religious, institutional, or other barriers for people to be VCA donors?

   [Transition: *Prior research studies suggest… A significantly larger proportion of the white (53.5%) and Asian (45.8%) populations were very willing to donate their hands compared with the African-American population (34.1%). When asked about donating their face, the African-American population (42.1%) was significantly more likely to report they were not at all willing to donate compared with the Caucasian (18.6%), Asian (18.9%), and Native American (21.4%) populations.*]
8. What may be some personal, financial, cultural, religious, institutional or other barriers for African American, Latino, or other groups to be VCA donors?
